# Feasibility and importance of universal suicide screening in a pediatric emergency department

**DOI:** 10.1371/journal.pone.0321934

**Published:** 2025-06-23

**Authors:** Steven C. Rogers, Shane J. Sacco, Kristen Volz, Danielle Chenard, Kevin Borrup, Kun Chen, Robert H. Aseltine

**Affiliations:** 1 Center for Population Health, UConn Health, Farmington, Connecticut, United States of America; 2 Connecticut Children’s Medical Center, Hartford, Connecticut, United States of America; 3 Department of Pediatrics, University of Connecticut School of Medicine, Farmington, Connecticut, United States of America; 4 Department of Statistics, University of Connecticut, Storrs, Connecticut, United States of America; 5 Department of Medicine, University of Connecticut School of Medicine, Farmington, Connecticut, United States of America; 6 Division of Behavioral Sciences and Community Health, UConn Health, Farmington, Connecticut, United States of America; NYU Grossman School of Medicine: New York University School of Medicine, UNITED STATES OF AMERICA

## Abstract

Suicide is a leading cause of death in the United States. In 2018, the Joint Commission recommended screening patients for suicide risk in healthcare settings. Universal screening may increase the safety of at-risk youth, but is challenging for many pediatric emergency departments. We examined the feasibility and outcomes associated with universal suicide risk screening using a combination of two screening tools in a pediatric emergency department. This retrospective cohort study examined 10–18 year old patients presenting to a large, urban pediatric emergency department between September 2019 and August 2021. Key variables included patient demographic information, suicide risk screening results, and subsequent suicide attempts. There were 30,328 encounters in the pediatric emergency department over this two-year period. Screening was completed 84.8% of the time. Of the 17,332 unique patients screened, 83.9% were at minimal suicide risk, 7.0% low risk, 2.1% moderate risk, and 7.0% high risk. In the 6 months following screening, low-risk patients returning to the emergency department were 7.1 times likely to have a suicide attempt than minimal-risk patients, moderate-risk patients were 9.8 times likely, and high-risk patients were 15.5 times likely. Universal screening in a pediatric emergency department is feasible and informative. The combined screening tool protocol appeared to enhance efficiency while maintaining clinical accuracy. Universal screening identified a substantial proportion of pediatric emergency department patients at risk of subsequent suicide attempts, with the likelihood of a subsequent attempt strongly linked to increasing risk levels identified by screening.

## Introduction

According to the Centers for Disease Control and Prevention (CDC), suicide is a leading cause of death in youth between the ages of 10 and 24 in the United States [1]. Despite many broad-based prevention efforts, death by suicide among children and young adults ages 12–17 in the US increased 47.7% between 2011–2022 [2]. Many youth who die by suicide have had a healthcare visit within months of their death, providing the impetus for improved early detection and intervention within healthcare systems [3,4]. Across all pediatric healthcare settings, the emergency department (ED) may offer the most opportune setting for detection and intervention, as it is the only contact with a healthcare provider for many youth and typically serves a population at high risk of suicidal behavior [5,6].

In recognition of this opportunity, the Joint Commission released National Patient Safety Goal (NPSG) 15.01.01 to improve the identification of patients at risk for suicide and the quality and safety of care while in healthcare settings [7]. The NPSG not only set standards for risk assessment and care, but also provided healthcare facilities with evidence-based screening tools that may be implemented to support screening efforts [8]. These tools include the Ask Suicide-Screening Questions (ASQ) tool and the Columbia Brief Suicide Severity Rating Scale (C-BSSRS), which are commonly used in pediatric settings [8,9]. Although it is difficult to demonstrate improved outcomes due to what remain nascent efforts to screen youth for suicide risk in pediatric EDs, universal screening has the potential to increase recognition of suicide risk by patients and families, decrease stigma by normalizing discussions about suicide, and identify pediatric EDs as a place that acts as a frontline for providing suicide-related care. Unfortunately, the limited behavioral health resources and high patient volumes in pediatric EDs yield it challenging to screen all incoming patients [10,11].

In this study, we present results from the implementation of a two-year universal suicide-screening program comprised of a clinical pathway that combined the ASQ and C-BSSRS in an urban pediatric ED serving a racially, ethnically, and socioeconomically diverse patient population. Our evaluation focused on rates of staff compliance, suicide risk screening results, and the association between screening risk level and rates of subsequent suicidal behaviors among patients returning to the ED.

## Methods

This study was approved by the Connecticut Children’s Medical Center Institutional Review Board.

### Cohort and dataset

In this retrospective study, clinical records were extracted from the electronic health records system (EPIC) between September 2019 (the initiation of the screening protocol) and March 2022. Records were delivered in an anonymous format and the only HIPAA identifiers were birth dates, service dates, and zip codes. A waiver of informed consent was obtained. We examined screening records between September 2019 and August 2021 (a two-year period), and examined diagnosis records through March 2022 (allowing for six months of follow-up) for the presence/absence of suicidal behaviors occurring after initial screening encounters. The study cohort included patients seen in the ED that were 10–18 years old during their screening encounter, or if missed by the screening protocol, during any point in the screening period.

### Measures

Demographic information included age (coded as 10–13 versus 14–18 years old), gender, self-reported race and ethnicity (coded as White, Black, Hispanic or Latino, Other race) and insurance payer (Medicaid versus other payment methods).

Suicide risk was assessed using a combination of two self-report questionnaires: the ASQ and C-BSSRS. The ASQ screening tool contains five items addressing suicidal thoughts and behaviors, and is validated for use in pediatric EDs [12–14]. The ASQ has a sensitivity of 96.7% and specificity of 91.1% in predicting results from a gold-standard measure of suicidal ideation, the Suicidal Ideation Questionnaire [13]. The C-BSSRS contains six items also addressing suicidal thoughts and behaviors, and is validated for detecting suicide risk, with sensitivities between 41.4%-53.9% and a specificity of 75.6% in predicting death by suicide within one week to a year of screening [15,16].

**Fig 1:**
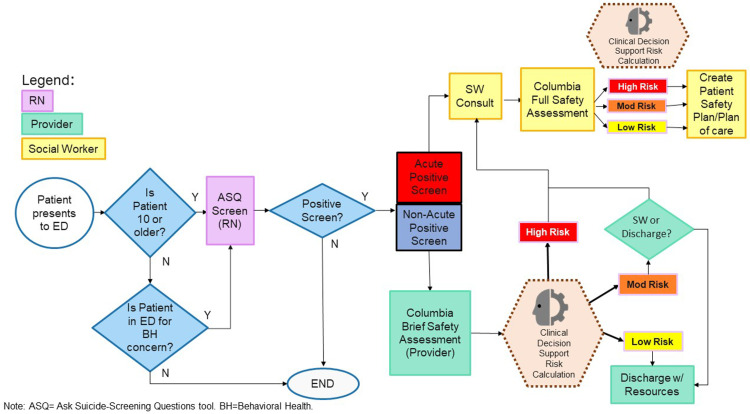
Suicide screening and assessments workflow.

An expert consensus pathway was developed specifically for use in EDs [17]. Our suicide screening clinical pathway used the ASQ to provide efficiencies in a busy clinical environment and the C-BSSRS to provide confirmation of risk and an expeditious way to further stratify risk levels among patients initially screening positive [12–17]. Patients deemed eligible for screening (i.e., medically stable and developmentally appropriate) were first administered the ASQ by nursing staff. If a patient was negative for risk or an acute positive, their final risk determination was minimal or high risk, respectively. Patients who were non-acute positive on the ASQ were then administered the C-BSSRS by provider staff to reaffirm positive risk and further stratify their risk level (low, moderate, high). Non-acute patients who either were low risk on the C-BSSRS, did not endorse “Yes” to any items on the C-BSSRS, or were missing their C-BSSRS, were assigned a final risk determination of low risk. Non-acute patients who were moderate or high risk on the C-BSSRS were assigned a final risk determination of moderate or high risk, respectively. See Fig 1 for full pathway details.

Suicide attempts within 6 months of screening were assessed via International Classification of Diseases version 10 (ICD-10) diagnosis codes. Given attempts are not defined clearly in the ICD-10 coding system [18] and the possible ambiguity in coding by physicians [19,20], we defined possible suicide attempts following precedents set by past studies [18,21–27]. Specifically, possible suicide attempts were coded as the presence/absence of codes indicating initial encounters for intentional self-harm (e.g., ICD-10 X60-X84), or combinations of suicide-related mental health disorder and injury codes in the same visit [22,24]. See S1 File for coding criteria and a full list of codes.

## Results

**Fig 2:**
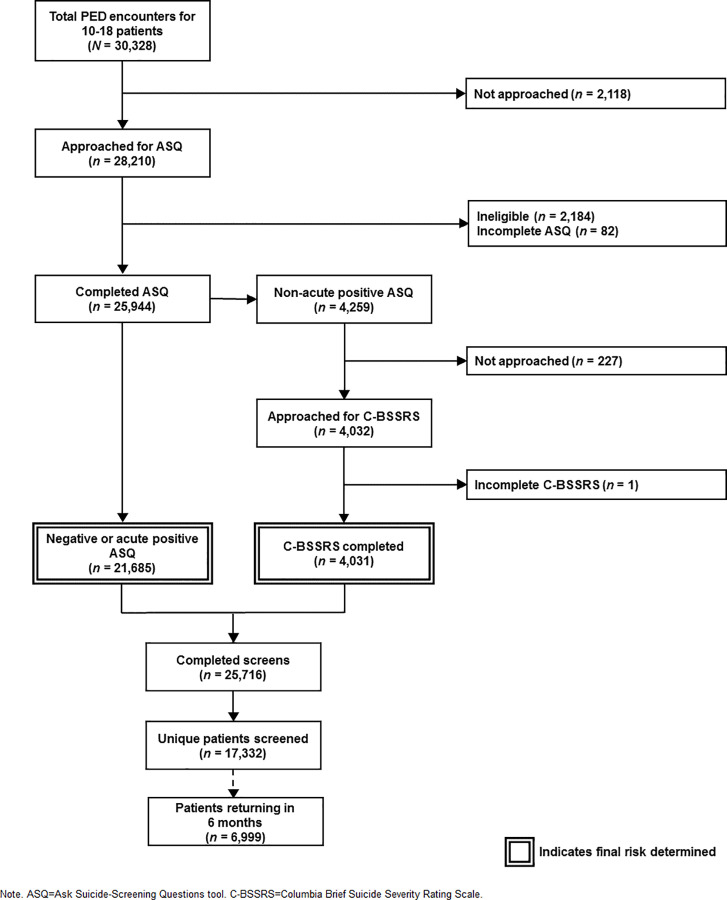
CONSORT diagram of completed screening protocol.

A patient flowchart summarizing screening completion rates and return rates within 6 months is presented in Fig 2. There were 30,328 ED encounters with 10–18 year old patients over the two-year screening period. Staff compliance with the screening protocol was excellent, with 93.0% (*n*=28,210) of eligible patients approached for screening. The overall completion rate of the ASQ at the encounter level was 85.5% (*n*=25,944). There were 4,259 (16.4%) patient encounters scoring as non-acute positive on the ASQ that were eligible for follow-up screening with the C-BSSRS. Provider staff completed the C-BSSRS for 94.7% (*n*=4,031) of these encounters. This yielded an overall completion rate of 84.8% (*n*=25,716).

**Fig 3:**
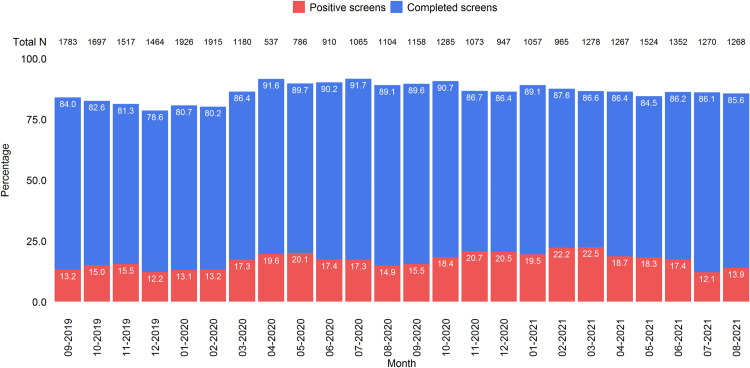
Screening encounters over the two years of data collection.

In Fig 3, we present a summary of completed screen rates and positive screen rates (i.e., at least a non-acute positive on the ASQ) for each month within the two-year screening period. The median volume of patients seen in the ED per month was 1,268 (minimum=537; maximum= 1,926). The median proportion of completed screens was 86.4% (minimum=78.6%; maximum= 91.7%), and the median proportion of positive screens was 17.3% (minimum=12.1%; maximum= 22.5%). Further Spearman’s correlation analysis revealed that compliance rates were higher when total ED volumes were lower (*ρ*=-0.71; *p*<0.001), and positive screen rates did not associate with total ED volume (*p*=0.17).

In Table 1, we summarize demographic characteristics and screening completion (i.e., completed screen, incomplete screen/ineligible, missed by protocol) among patients at their initial/only screening encounter. Of the 19,653 unique patients seen in the ED about half of patients were 14–18 years old, White race, female sex, and had Medicaid insurance. Most patients had at least one completed screen (88.2%; *n*=17,332). Few patients had only an incomplete screen or were ineligible (5.9%; *n*=1,163) or were missed by the protocol (5.9%; *n*=1,158). Based upon chi-square tests with *p*-values adjusted using Bonferroni correction, patients with completed screens were older age (91.5% among 14–18 versus 84.4% 10–13 years old) and female sex (90.2% versus 86.2% for male sex), *p*s<0.001. Screening completion rates also differed by self-reported race and ethnicity, such that, patients identifying as “Other race” were less likely to complete screening, *p*=0.02. Screening completion rates did not differ for White, Black, Hispanic or Latino race patients (*p*s>0.07), nor differ by insurance payer (*p*=0.82).

**Table 1:** Cohort characteristics.

Variable	Total cohort		Completed screen		Incomplete/ineligible		Missed by protocol		Group comparison	
	%	*N*	%	*n*	%	*n*	%	*n*	χ^2^	*p*
**No of patients**	100.0	19,653	88.2	17,332	5.9	1,163	5.9	1,158		
**Age in years**										
** 10-13**	47.0	9,247	84.4	7,807	8.5	790	7.0	650	269.8	<0.001
** 14-18**	53.0	10,406	91.5	9,525	3.6	373	4.9	508		
**Gender**										
** Male**	49.1	9,646	86.2	8,311	7.1	683	6.8	652	76.3	<0.001
** Female**	50.9	10,007	90.2	9,021	4.8	480	5.1	506		
**Race and ethnicity**									15.6	0.02
** White**	39.9	7,839	88.7	6,952	5.9	462	5.4	425		
** Black**	15.8	3,111	87.6	2,724	5.8	180	6.7	207		
** Hispanic or Latino**	33.9	6,655	88.5	5,890	5.8	387	5.7	378		
** Other race**	10.4	2,048	86.2	1,766	6.5	134	7.2	148		
**Medicaid**										
** Yes**	53.3	10,476	88.2	9,240	6.0	627	5.8	609	0.4	0.82
** No**	46.7	9,177	88.2	8,092	5.8	536	6.0	549		

**Fig 4:**
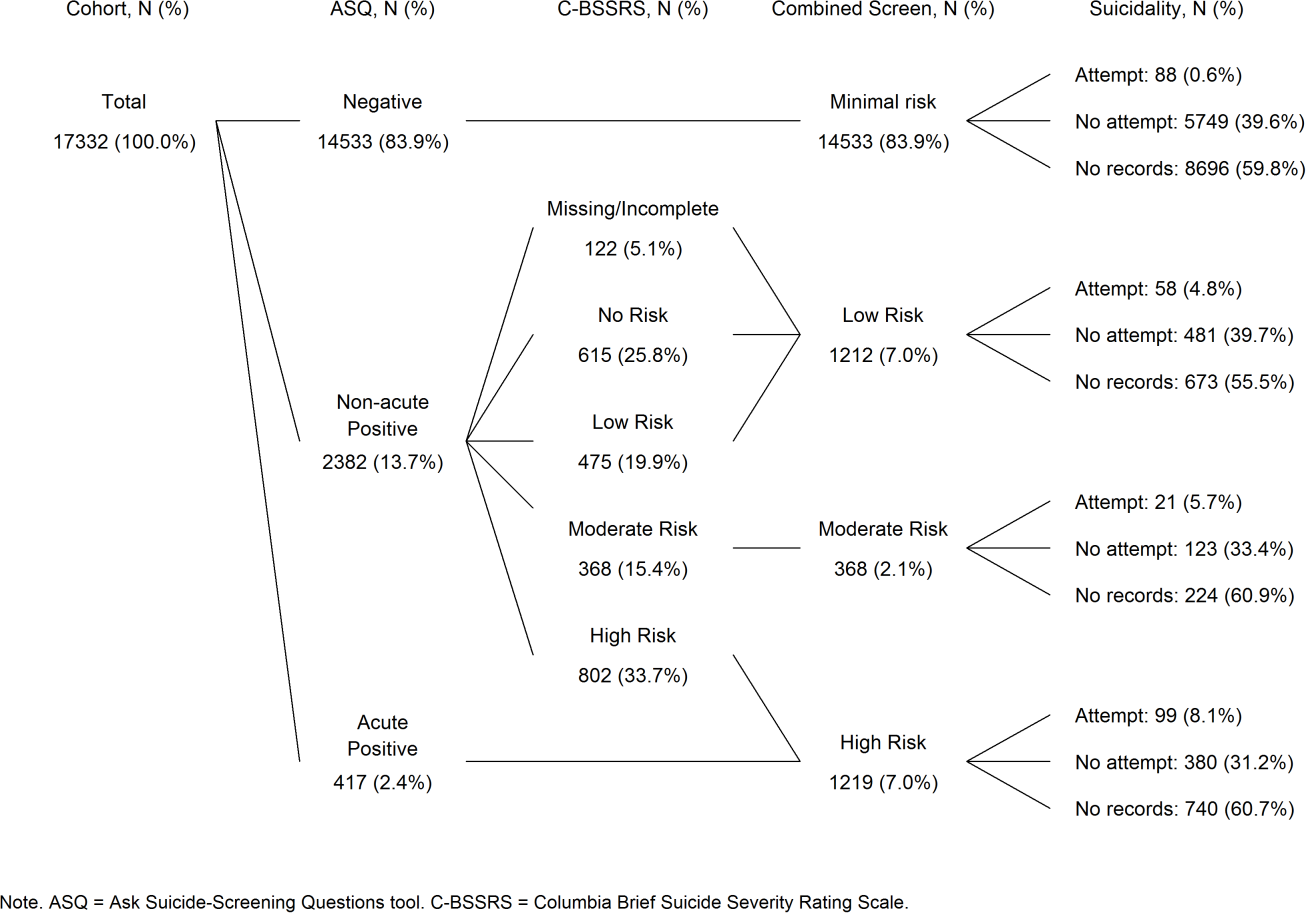
Flow chart presenting results from the combined ASQ/C-BSSRS suicide risk screening protocol.

In Fig 4, we summarize suicide risk results from the screening protocol. Of the 17,332 unique patients with completed screens, 83.9% received a negative score and 2.4% received an acute positive score on the ASQ, yielding final risk determinations of minimal and high risk, respectively. Around 14% of screened patients received a non-acute positive score on the ASQ and received a follow-up risk assessment with the C-BSSRS. Among these patients, 45.7% were classified as low or no risk, 15.4% as moderate risk, and 33.7% as high risk. About 5% of non-acute positives on the ASQ did not complete a C-BSSRS. Combining the results across both screening instruments (and classifying non-acute positives on the ASQ who were missing the C-BSSRS as low risk) yielded a total of 83.9% patients designated as minimal risk, 7.0% low risk, 2.1% moderate risk, and 7.0% high risk (Fig 4, 4^th^ column from the left).

In Table 2, we present differences in suicide risk screening results among demographic groups. Statistically significant differences in screening results were observed across all demographic characteristics based on chi-square tests with *p*-values adjusted using Bonferroni correction, although most of these differences were small to modest in magnitude (Multiple comparison results are presented in S1 Table). Older age, female sex, and White race patients were more likely to be designated as higher risk than were younger age, male sex, and non-White race patients, respectively, *p*s<0.001. Medicaid patients were slightly less likely to be designated as higher risk than commercially insured or non-insured patients, *p*<0.001.

**Table 2:** Final risk determination by demographic characteristics.

Variable	Final risk determination									
	Minimal		Low		Moderate		High		Group comparison	
	%	*n*	%	*n*	%	*n*	%	*n*	χ^2^	*p*
**No of patients**	83.9	14,533	7.0	1,212	2.1	368	7.0	1,219		
**Age in years**										
** 10-13**	87.3	6,776	5.6	437	1.5	117	5.6	436	122.4	<0.001
** 14-18**	81.1	7,757	8.1	775	2.6	251	8.2	783		
**Gender**										
** Male**	89.8	7,466	4.9	404	1.4	114	3.9	327	432.4	<0.001
** Female**	78.3	7,067	9.0	808	2.8	254	9.9	892		
**Race and ethnicity**									112.5	<0.001
** White**	81.2	5,642	7.3	509	2.6	183	8.9	618		
** Black**	86.1	2,346	6.9	189	1.6	43	5.4	146		
** Hispanic or Latino**	86.4	5,086	6.8	400	1.7	100	5.2	304		
** Other race**	82.6	1,459	6.5	114	2.4	42	8.6	151		
**Medicaid**										
** Yes**	84.1	7,776	7.6	702	2.1	189	6.2	575	29.6	<0.001
** No**	83.5	6,757	6.3	510	2.2	179	8.0	644		

In Table 3, we provide results from a logistic regression analysis assessing the likelihood, as odds, of a patient returning within 6 months of screening with a suicide attempt, given suicide risk level and controlling for demographic variables. Note that more than half of patients did not have a follow-up ED visit within 6 months of the screening encounter (*n*=10,333) and that the numbers of patients without subsequent ED visits varied minimally across the four risk levels based on a chi-square test, χ^2^(3, *n*=17,322)=9.55, *p*=0.02. See S2 Table for multiple comparisons with *p*-values adjusted using Bonferroni correction. Among patients with subsequent ED visits (*n*=6,999), rates of suicide attempts differed substantially by risk level. Compared to minimal-risk patients, low-risk patients were 7.1 times likely to have an attempt, moderate-risk patients were 9.8 times likely to have an attempt, and high-risk patients were between 15.5 times likely to have an attempt. Converting the odds to proportions, these data indicate that among those returning to the ED, 1.5% of minimal-risk patients, 9.6% of low-risk patients, 12.5% of moderate-risk patients, and 18.8% of high-risk patients were treated for a subsequent suicide attempt. Further chi-square analysis contrasting patients classified as acute positives on the ASQ and those classified as high risk on the C-BSSRS revealed that these two high-risk groups did not differ significantly in their likelihood of a subsequent suicide attempt, *p*=0.44.

**Table 3:** Logistic regression analysis presenting associations between suicide attempts and risk level, controlling for demographic characteristics.

Variable	Odds [95% CI]	*z*	*p*
**Intercept**	0.01 [0.01, 0.02]	-23.97	<0.001
**Screening result**			
** Minimal risk**	Reference		
** Low risk**	7.11 [4.62, 9.59]	11.00	<0.001
** Moderate risk**	9.78 [4.72, 14.85]	8.63	<0.001
** High risk**	15.47 [10.62, 20.32]	17.11	<0.001
**Age (in years)**			
** 10–13**	Reference		
** 14–18**	0.98 [0.72, 1.23]	-0.19	0.85
**Gender**			
** Male**	Reference		
** Female**	1.53 [1.08, 1.97]	2.87	0.004
**Race and ethnicity**			
** White**	Reference		
** Black**	0.64 [0.37, 0.90]	-2.12	0.03
** Hispanic or Latino**	0.69 [0.47, 0.92]	-2.21	0.03
** Other race**	0.75 [0.40, 1.10]	-1.20	0.23
**Medicaid**			
** No**	Reference		
** Yes**	1.64 [1.17, 2.12]	3.36	0.001

To further examine the degree to which screening results predicted subsequent attempts among patients with follow-up ED encounters, we calculated sensitivities, positive predictive values (PPV), and specificities when varying the cut-off of suicide risk screening results (i.e., any at-risk category, moderate or high risk, high risk only). See Table 4. Screening identified 66.9% of patients returning with an attempt given any at-risk category, 45.1% given moderate or high risk, and 37.2% given high risk only. Patients were accurately labeled at risk (i.e., had PPV of) 15.3% of the time given any at-risk category, 19.3% given moderate or high risk, and 20.7% given high risk only. The specificity (i.e., the percent of patients accurately identified as no risk) increased given more restrictive risk cut-off points, from 85.4% to 94.4%.

**Table 4:** Predicting suicide attempts given varied risk cut-offs.

Risk cut-off	Sensitivity	PPV	Specificity
**Any at-risk category**	66.9	15.3	85.4
**Moderate or high risk**	45.1	19.3	92.5
**High risk only**	37.2	20.7	94.4

Note. PPV=Positive predictive value.

### Post hoc analysis

Because this analysis draws on data from a single hospital, we could not rule out the possibility that patients at this site had visited other hospitals in the region following their screening encounter. To address this question, we examined statewide hospital claims data from 2012–2017 to examine the extent to which ED patients from this facility used other facilities. For a fair comparison, we matched the lengths of the recruitment and follow-up periods (two years and six months, respectively). Out of the 20,197 patients in this prior cohort, the vast majority of patients (92.6%; *n*=18,706) had either no follow-up visit (65.2%; *n*=13,178) or returned only to this hospital (27.4%; *n*=5,528).

## Discussion

This study of patients from a large urban setting provides compelling evidence of both the feasibility and clinical value of universal suicide risk screening in the pediatric ED. First, our results demonstrated that suicide risk screening using validated screening tools can be implemented and sustained with high rates of staff adherence in a busy ED environment. Our screening efforts identified a substantial proportion of at-risk patients (16.1%), similar to other studies of pediatric ED patients [28,29]. Of critical importance, our data confirmed that risk stratification by screening tools have prognostic value based upon the association between risk levels and subsequent suicide events, where 18.8% of high-risk patients and 12.5% of moderate-risk patients had a follow-up ED encounter involving a suicide attempt within 6 months of screening, compared to only 1.5% of minimal-risk patients.

These results also revealed the advantages of a screening protocol that combined the ASQ and C-BSSRS to maximize efficiency and accuracy in a busy clinical environment. The ASQ is preferred by clinical staff for suicide risk screening given its efficiencies including limited time to complete and ease of scoring [12,13]. However, our analysis suggests that further discrimination among ASQ non-acute positives was necessary, as over one-third of these patients were actually classified as high risk by C-BSSRS. The approach used in this study, which was based on the consensus guideline published in 2019 by Brahmbhatt et al. reduced the added burden of screening using the longer battery of questions in the C-BSSRS to only 14% of the overall ED population [17]. Note that we found no differences in the likelihood of a subsequent suicide attempt among patients classified as acute positives on the ASQ and those classified as high-risk on the C-BSSRS, providing further justification for combining the results from these screening tools.

However, our results also showed that assumptions regarding the clinical needs of those deemed to be at low risk may need revision. These patients were at 7 times the risk of an encounter involving a suicide attempt within 6 months of screening, suggesting that a greater clinical response or intervention may be necessary with this subset of patients, beyond providing community-based resources (e.g., a more formal clinical assessment; referral to behavioral health provider; safety planning; scheduled follow-up visits). This finding has implications for a facility’s assessment of the feasibility of universal screening, and allocated resources required to support this effort. In our study, providing additional resources/intervention to the over 1,200 low-risk patients would have nearly doubled the size of the at-risk group to almost 17% of the total patient population. Addressing the needs of this larger patient population may be impractical given the dearth of mental and behavioral health resources in most EDs [10,30]. When developing a suicide prevention program, EDs should consider available institutional resources, partnerships with community mental health providers and other potential services such as telehealth to meet the needs of those who screen positive for risk.

### Limitations

There are several limitations to this analysis. While providing services to a large and racially, ethnically, and socioeconomically diverse population, it is a single institution in an urban setting, which may limit broader applications. For instance, results may have differed in smaller hospitals with more limited resources (e.g., rural settings), hospitals with differing demographic distributions (e.g., we noted some differences in suicide screen completion by age and gender), or hospitals with more restrictive regulatory policies or guidelines (e.g., triage staff may not be allowed to assess mental/behavioral health concerns using suicide screens or other means). Subsequently, replicating these findings in future work may help further assess generalizability to other facilities and populations. We noted slightly lower rates of suicide screen completion in 10–13 year old patients and male sex patients, which may indicate limitations in our protocol and/or risk screening in general. Given this was a retrospective study and we could not collect quality improvement data, future research should explore this phenomenon further, addressing potential causes of this disparity and solution strategies (e.g., different administration modalities). Finally, although this pediatric ED is the largest provider of emergency mental/behavioral healthcare in the state and a substantial number of patients returned to the ED within 6 months of screening, we did not have access to patient data/encounters outside of this facility. Thus, it is unclear if results would have differed given inclusion of other hospital data. However, our analysis of statewide hospital claims data found that only 8% of ED patients at this facility presented to a different facility within 6 months of an encounter, minimizing this concern.

## Conclusion

Universal suicide risk screening for youth in a pediatric ED environment is both feasible and clinically informative. Use of a screening protocol that combines the ASQ and C-BSSRS appears to enhance efficiency while maintaining clinical accuracy and may be particularly helpful in busy clinical settings. However, the high prevalence of suicide risk among children presenting to the ED may pose challenges in clinical settings with limited behavioral health resources. Identifying all at-risk youth and offering them resources prior to a suicide attempt may ultimately improve patient safety but is likely to be costly and resource intensive when the at-risk group approaches 17% of patients. In addition, the high prevalence of ED visits related to potential suicidal behaviors within 6 months of a positive screening for suicide risk is sobering and suggests that interventions deployed in the ED for those identified as at-risk may have limited efficacy. Further research to develop and improve best practices in identifying youth at risk of suicide and connecting these youth to effective care is essential.

## Supporting information

S1 File Suicide behaviors identification algorithm rules and code list. (PDF)

S1 Table Comparisons of patient characteristics by risk level. (PDF)

S2 Table Comparison of return rates in 6 months by risk level. (PDF)
