# Supplementary material for: Feasibility and importance of universal suicide screening in a pediatric emergency department
Source: PLoS One. 2025 Jun 23;20(6):e0321934. doi: 10.1371/journal.pone.0321934 (PMC12184887; doi:10.1371/journal.pone.0321934)
Supplement: S1 File — (PDF) [file pone.0321934.s001.pdf]

S1 File. Suicide behaviors identification algorithm rules and code list.

Identification algorithm rules

Rule	ICD-10 Code Categories
Intentional self-harm	1. Suicide attempt OR<br>2. Intentional self-harm events OR<br>3. Poisoning and toxic effects, intentional self-harm OR<br>4. Asphyxiation, intentional self-harm
Ideation and Injuries or Events	1a. Suicidal ideation<br><br>AND in the same visit:<br><br>1b. Events of undetermined intent OR<br>1c. Poisoning and toxic effects, undetermined intent OR<br>1d. Asphyxiation, undetermined intent OR<br>1e. Injuries including lacerations, contusions, abrasions, wounds, penetration, puncturing, and superficial foreign bodies and injuries
Suicide-related mental disorders and Suicide-related Injuries or Events	1a. Mental health disorders including depression, dysthymia, mania, bipolar, schizophrenia, behavioral disturbances, anxiety (excluding phobias and separation), stress, delusions, psychotic states, and unspecified mental disorders<br><br>AND in the same visit:<br><br>1b. Events of undetermined intent OR<br>1c. Poisoning and toxic eff, undetermined intent OR<br>1d. Asphyxiation, undetermined intent OR<br>1e. Injuries to wrists, head, or neck including lacerations, contusions, abrasions, wounds, penetration, puncturing, and superficial foreign bodies and injuries
Note. To add certainty that coded attempts were not consequential of prior encounters, we only included injury codes specifying that they were newly occurring (i.e., “initial encounter”; code extension A) and not follow-up care (i.e., “subsequent encounter”; code extension D) or complications related to an initial injury (i.e., “sequelae”; code extension S).	

Full list of ICD-10 codes for suicide attempt identification

Rule	Category	Codes
Rule 1	Suicide attempt, initial encounter	T1491XA
	Event, intentional self-harm, initial encounter	X710XXA; X711XXA; X712XXA; X713XXA; X718XXA; X719XXA; X72XXXA; X730XXA; X731XXA; X732XXA; X738XXA; X739XXA; X7401XA; X7402XA; X7409XA; X748XXA; X749XXA; X75XXXA; X76XXXA; X770XXA; X771XXA; X772XXA; X773XXA; X778XXA; X779XXA; X780XXA; X781XXA; X782XXA; X788XXA; X789XXA; X79XXXA; X80XXXA; X810XXA; X811XXA; X818XXA; X828XXA; X830XXA; X831XXA; X832XXA; X838XXA
	Poisoning, intentional self-harm, initial encounter	T360X2A; T361X2A; T362X2A; T363X2A; T364X2A; T365X2A; T366X2A; T367X2A; T368X2A; T3692XA; T370X2A; T371X2A; T372X2A; T373X2A; T374X2A; T375X2A; T378X2A; T3792XA; T380X2A; T381X2A; T382X2A; T383X2A; T384X2A; T385X2A; T386X2A; T387X2A; T38802A; T38812A; T38892A; T38902A; T38992A; T39012A; T39092A; T391X2A; T392X2A; T39312A; T39392A; T394X2A; T398X2A; T3992XA; T400X2A; T401X2A; T402X2A; T403X2A; T404X2A; T405X2A; T40602A; T40692A; T407X2A; T408X2A; T40902A; T40992A; T410X2A; T411X2A; T41202A; T41292A; T413X2A; T4142XA; T415X2A; T420X2A; T421X2A; T422X2A; T423X2A; T424X2A; T425X2A; T426X2A; T4272XA; T428X2A; T43012A; T43022A; T431X2A; T43202A; T43212A; T43222A; T43292A; T433X2A; T434X2A; T43502A; T43592A; T43602A; T43612A; T43622A; T43632A; T43692A; T438X2A; T4392XA; T440X2A; T441X2A; T442X2A; T443X2A; T444X2A; T445X2A; T446X2A; T447X2A; T448X2A; T44902A; T44992A; T450X2A; T451X2A; T452X2A; T453X2A; T454X2A; T45512A; T45522A; T45602A; T45612A; T45622A; T45692A; T457X2A; T458X2A; T4592XA; T460X2A; T461X2A; T462X2A; T463X2A; T464X2A; T465X2A; T466X2A; T467X2A; T468X2A; T46902A; T46992A; T470X2A; T471X2A; T472X2A; T473X2A; T474X2A; T475X2A; T476X2A; T477X2A; T478X2A; T4792XA; T480X2A; T481X2A; T48202A; T48292A; T483X2A; T484X2A; T485X2A; T486X2A; T48902A; T48992A; T490X2A; T491X2A; T492X2A; T493X2A; T494X2A; T495X2A; T496X2A; T497X2A; T498X2A; T4992XA; T500X2A; T501X2A; T502X2A; T503X2A; T504X2A; T505X2A; T506X2A; T507X2A;

		T508X2A; T50A12A; T50A22A; T50A92A; T50B12A; T50B92A; T50Z12A; T50Z92A; T50902A; T50992A
	Toxic effect, intentional self-harm, initial encounter	T510X2A; T511X2A; T512X2A; T513X2A; T518X2A; T5192XA; T520X2A; T521X2A; T522X2A; T523X2A; T524X2A; T528X2A; T5292XA; T530X2A; T531X2A; T532X2A; T533X2A; T534X2A; T535X2A; T536X2A; T537X2A; T5392XA; T540X2A; T541X2A; T542X2A; T543X2A; T5492XA; T550X2A; T551X2A; T560X2A; T561X2A; T562X2A; T563X2A; T564X2A; T565X2A; T566X2A; T567X2A; T56812A; T56892A; T5692XA; T570X2A; T571X2A; T572X2A; T573X2A; T578X2A; T5792XA; T5802XA; T5812XA; T582X2A; T588X2A; T5892XA; T590X2A; T591X2A; T592X2A; T593X2A; T594X2A; T595X2A; T596X2A; T597X2A; T59812A; T59892A; T5992XA
	Asphyxiation, intentional self-harm, initial encounter	T71112A; T71122A; T71132A; T71152A; T71162A; T71192A; T71222A; T71232A
Rule 2A	Suicide ideation, initial encounter	R45851
Rule 2B	Event, undetermined intent, initial encounter	Y210XXA; Y211XXA; Y212XXA; Y213XXA; Y214XXA; Y218XXA; Y219XXA; Y22XXXA; Y230XXA; Y231XXA; Y232XXA; Y233XXA; Y238XXA; Y239XXA; Y240XXA; Y248XXA; Y249XXA; Y25XXXA; Y26XXXA; Y270XXA; Y271XXA; Y272XXA; Y273XXA; Y278XXA; Y279XXA; Y280XXA; Y281XXA; Y282XXA; Y288XXA; Y289XXA; Y29XXXA; Y30XXXA; Y31XXXA; Y32XXXA; Y33XXXA
	Poison, undetermined intent, initial encounter	T360X4A; T361X4A; T362X4A; T363X4A; T364X4A; T365X4A; T366X4A; T367X4A; T368X4A; T3694XA; T370X4A; T371X4A; T372X4A; T373X4A; T374X4A; T375X4A; T378X4A; T3794XA; T380X4A; T381X4A; T382X4A; T383X4A; T384X4A; T385X4A; T386X4A; T387X4A; T38804A; T38814A; T38894A; T38904A; T38994A; T39014A; T39094A; T391X4A; T392X4A; T39314A; T39394A; T394X4A; T398X4A; T3994XA; T400X4A; T401X4A; T402X4A; T403X4A; T404X4A; T405X4A; T40604A; T40694A; T407X4A; T408X4A; T40904A; T40994A; T410X4A; T411X4A; T41204A; T41294A; T413X4A; T4144XA; T415X4A; T420X4A; T421X4A; T422X4A; T423X4A; T424X4A; T425X4A; T426X4A; T4274XA; T428X4A; T43014A; T43024A; T431X4A; T43204A; T43214A; T43224A; T43294A;

		T433X4A; T434X4A; T43504A; T43594A; T43604A; T43614A; T43624A; T43634A; T43694A; T438X4A; T4394XA; T440X4A; T441X4A; T442X4A; T443X4A; T444X4A; T445X4A; T446X4A; T447X4A; T448X4A; T44904A; T44994A; T450X4A; T451X4A; T452X4A; T453X4A; T454X4A; T45514A; T45524A; T45604A; T45614A; T45624A; T45694A; T457X4A; T458X4A; T4594XA; T460X4A; T461X4A; T462X4A; T463X4A; T464X4A; T465X4A; T466X4A; T467X4A; T468X4A; T46904A; T46994A; T470X4A; T471X4A; T472X4A; T473X4A; T474X4A; T475X4A; T476X4A; T477X4A; T478X4A; T4794XA; T480X4A; T481X4A; T48204A; T48294A; T483X4A; T484X4A; T485X4A; T486X4A; T48904A; T48994A; T490X4A; T491X4A; T492X4A; T493X4A; T494X4A; T495X4A; T496X4A; T497X4A; T498X4A; T4994XA; T500X4A; T501X4A; T502X4A; T503X4A; T504X4A; T505X4A; T506X4A; T507X4A; T508X4A; T50A14A; T50A24A; T50A94A; T50B14A; T50B94A; T50Z14A; T50Z94A; T50904A; T50994A
	Toxic effect, undetermined intent, initial encounter	T510X4A; T511X4A; T512X4A; T513X4A; T518X4A; T5194XA; T520X4A; T521X4A; T522X4A; T523X4A; T524X4A; T528X4A; T5294XA; T530X4A; T531X4A; T532X4A; T533X4A; T534X4A; T535X4A; T536X4A; T537X4A; T5394XA; T540X4A; T541X4A; T542X4A; T543X4A; T5494XA; T550X4A; T551X4A; T560X4A; T561X4A; T562X4A; T563X4A; T564X4A; T565X4A; T566X4A; T567X4A; T56814A; T56894A; T5694XA; T570X4A; T571X4A; T572X4A; T573X4A; T578X4A; T5794XA; T5804XA; T5814XA; T582X4A; T588X4A; T5894XA; T590X4A; T591X4A; T592X4A; T593X4A; T594X4A; T595X4A; T596X4A; T597X4A; T59814A; T59894A; T5994XA
	Asphyxiation, undetermined intent, initial encounter	T71114A; T71124A; T71134A; T71144A; T71154A; T71164A; T71194A; T71224A; T71234A
	Contusion, laceration, and hemorrhage, initial encounter	S06370A; S06371A; S06372A; S06373A; S06374A; S06375A; S06376A; S06377A; S06378A; S06379A; S06380A; S06381A; S06382A; S06383A; S06384A; S06385A; S06386A; S06387A; S06388A; S06389A
	Contusion and Laceration, initial encounter	S06310A; S06311A; S06312A; S06313A; S06314A; S06315A; S06316A; S06317A; S06318A; S06319A; S06320A; S06321A; S06322A; S06323A; S06324A; S06325A; S06326A; S06327A;

		S06328A; S06329A; S06330A; S06331A; S06332A; S06333A; S06334A; S06335A; S06336A; S06337A; S06338A; S06339A
	Laceration, initial encounter	S0101XA; S0102XA; S01111A; S01112A; S01119A; S01121A; S01122A; S01129A; S0121XA; S0122XA; S01311A; S01312A; S01319A; S01321A; S01322A; S01329A; S01411A; S01412A; S01419A; S01421A; S01422A; S01429A; S01511A; S01512A; S01521A; S01522A; S0181XA; S0182XA; S0191XA; S0192XA; S0520XA; S0521XA; S0522XA; S0530XA; S0531XA; S0532XA; S0912XA; S11011A; S11012A; S11021A; S11022A; S11031A; S11032A; S1111XA; S1112XA; S1121XA; S1122XA; S1181XA; S1182XA; S1191XA; S1192XA; S15011A; S15012A; S15019A; S15021A; S15022A; S15029A; S15111A; S15112A; S15119A; S15121A; S15122A; S15129A; S15211A; S15212A; S15219A; S15221A; S15222A; S15229A; S15311A; S15312A; S15319A; S15321A; S15322A; S15329A; S162XXA; S21011A; S21012A; S21019A; S21021A; S21022A; S21029A; S21111A; S21112A; S21119A; S21121A; S21122A; S21129A; S21211A; S21212A; S21219A; S21221A; S21222A; S21229A; S21311A; S21312A; S21319A; S21321A; S21322A; S21329A; S21411A; S21412A; S21419A; S21421A; S21422A; S21429A; S2191XA; S2192XA; S2501XA; S2502XA; S25111A; S25112A; S25119A; S25121A; S25122A; S25129A; S2521XA; S2522XA; S25311A; S25312A; S25319A; S25321A; S25322A; S25329A; S25411A; S25412A; S25419A; S25421A; S25422A; S25429A; S25511A; S25512A; S25519A; S25811A; S25812A; S25819A; S2591XA; S26020A; S26021A; S26022A; S2612XA; S2692XA; S27331A; S27332A; S27339A; S27431A; S27432A; S27439A; S2753XA; S2763XA; S27803A; S27813A; S27893A; S29021A; S29022A; S29029A; S31010A; S31011A; S31020A; S31021A; S31110A; S31111A; S31112A; S31113A; S31114A; S31115A; S31119A; S31120A; S31121A; S31122A; S31123A; S31124A; S31125A; S31129A; S3121XA; S3122XA; S3131XA; S3132XA; S3141XA; S3142XA; S31511A; S31512A; S31521A; S31522A; S31610A; S31611A; S31612A; S31613A; S31614A; S31615A; S31619A; S31620A; S31621A; S31622A; S31623A; S31624A; S31625A; S31629A; S31801A; S31802A; S31811A; S31812A; S31821A; S31822A; S31831A; S31832A; S3501XA; S3502XA; S3511XA; S3512XA; S35211A; S35212A; S35221A; S35222A; S35231A; S35232A; S35291A; S35292A; S35311A; S35321A; S35331A; S35341A; S35411A; S35412A; S35413A; S35414A; S35415A; S35416A; S358X1A; S3591XA; S36030A; S36031A; S36032A; S36039A; S36113A; S36114A; S36115A; S36116A; S36123A;

		S36230A; S36231A; S36232A; S36239A; S36240A; S36241A; S36242A; S36249A; S36250A; S36251A; S36252A; S36259A; S36260A; S36261A; S36262A; S36269A; S3633XA; S36430A; S36438A; S36439A; S36530A; S36531A; S36532A; S36533A; S36538A; S36539A; S3663XA; S36893A; S3693XA; S37031A; S37032A; S37039A; S37041A; S37042A; S37049A; S37051A; S37052A; S37059A; S37061A; S37062A; S37069A; S3713XA; S3723XA; S3733XA; S37431A; S37432A; S37439A; S37531A; S37532A; S37539A; S3763XA; S37813A; S37823A; S37893A; S3793XA; S39021A; S39022A; S39023A; S41011A; S41012A; S41019A; S41021A; S41022A; S41029A; S41111A; S41112A; S41119A; S41121A; S41122A; S41129A; S45011A; S45012A; S45019A; S45111A; S45112A; S45119A; S45211A; S45212A; S45219A; S45311A; S45312A; S45319A; S45811A; S45812A; S45819A; S45911A; S45912A; S45919A; S46021A; S46022A; S46029A; S46121A; S46122A; S46129A; S46221A; S46222A; S46229A; S46321A; S46322A; S46329A; S46821A; S46822A; S46829A; S46921A; S46922A; S46929A; S51011A; S51012A; S51019A; S51021A; S51022A; S51029A; S51811A; S51812A; S51819A; S51821A; S51822A; S51829A; S55011A; S55012A; S55019A; S55111A; S55112A; S55119A; S55211A; S55212A; S55219A; S55811A; S55812A; S55819A; S55911A; S55912A; S55919A; S56021A; S56022A; S56029A; S56121A; S56122A; S56123A; S56124A; S56125A; S56126A; S56127A; S56128A; S56129A; S56221A; S56222A; S56229A; S56321A; S56322A; S56329A; S56421A; S56422A; S56423A; S56424A; S56425A; S56426A; S56427A; S56428A; S56429A; S56521A; S56522A; S56529A; S56821A; S56822A; S56829A; S56921A; S56922A; S56929A; S61011A; S61012A; S61019A; S61021A; S61022A; S61029A; S61111A; S61112A; S61119A; S61121A; S61122A; S61129A; S61210A; S61211A; S61212A; S61213A; S61214A; S61215A; S61216A; S61217A; S61218A; S61219A; S61220A; S61221A; S61222A; S61223A; S61224A; S61225A; S61226A; S61227A; S61228A; S61229A; S61310A; S61311A; S61312A; S61313A; S61314A; S61315A; S61316A; S61317A; S61318A; S61319A; S61320A; S61321A; S61322A; S61323A; S61324A; S61325A; S61326A; S61327A; S61328A; S61329A; S61411A; S61412A; S61419A; S61421A; S61422A; S61429A; S61511A; S61512A; S61519A; S61521A; S61522A; S61529A; S65011A; S65012A; S65019A; S65111A; S65112A; S65119A; S65211A; S65212A; S65219A; S65311A; S65312A; S65319A; S65411A; S65412A; S65419A; S65510A; S65511A; S65512A; S65513A; S65514A; S65515A; S65516A; S65517A; S65518A; S65519A; S65811A; S65812A; S65819A; S65911A; S65912A; S65919A; S66021A; S66022A; S66029A; S66120A; S66121A; S66122A; S66123A;

		S66124A; S66125A; S66126A; S66127A; S66128A; S66129A; S66221A; S66222A; S66229A; S66320A; S66321A; S66322A; S66323A; S66324A; S66325A; S66326A; S66327A; S66328A; S66329A; S66421A; S66422A; S66429A; S66520A; S66521A; S66522A; S66523A; S66524A; S66525A; S66526A; S66527A; S66528A; S66529A; S66821A; S66822A; S66829A; S66921A; S66922A; S66929A; S71011A; S71012A; S71019A; S71021A; S71022A; S71029A; S71111A; S71112A; S71119A; S71121A; S71122A; S71129A; S75011A; S75012A; S75019A; S75021A; S75022A; S75029A; S75111A; S75112A; S75119A; S75121A; S75122A; S75129A; S75211A; S75212A; S75219A; S75221A; S75222A; S75229A; S75811A; S75812A; S75819A; S75911A; S75912A; S75919A; S76021A; S76022A; S76029A; S76121A; S76122A; S76129A; S76221A; S76222A; S76229A; S76321A; S76322A; S76329A; S76821A; S76822A; S76829A; S76921A; S76922A; S76929A; S81011A; S81012A; S81019A; S81021A; S81022A; S81029A; S81811A; S81812A; S81819A; S81821A; S81822A; S81829A; S85011A; S85012A; S85019A; S85111A; S85112A; S85119A; S85141A; S85142A; S85149A; S85171A; S85172A; S85179A; S85211A; S85212A; S85219A; S85311A; S85312A; S85319A; S85411A; S85412A; S85419A; S85511A; S85512A; S85519A; S85811A; S85812A; S85819A; S85911A; S85912A; S85919A; S86021A; S86022A; S86029A; S86121A; S86122A; S86129A; S86221A; S86222A; S86229A; S86321A; S86322A; S86329A; S86821A; S86822A; S86829A; S86921A; S86922A; S86929A; S91011A; S91012A; S91019A; S91021A; S91022A; S91029A; S91111A; S91112A; S91113A; S91114A; S91115A; S91116A; S91119A; S91121A; S91122A; S91123A; S91124A; S91125A; S91126A; S91129A; S91211A; S91212A; S91213A; S91214A; S91215A; S91216A; S91219A; S91221A; S91222A; S91223A; S91224A; S91225A; S91226A; S91229A; S91311A; S91312A; S91319A; S91321A; S91322A; S91329A; S95011A; S95012A; S95019A; S95111A; S95112A; S95119A; S95211A; S95212A; S95219A; S95811A; S95812A; S95819A; S95911A; S95912A; S95919A; S96021A; S96022A; S96029A; S96121A; S96122A; S96129A; S96221A; S96222A; S96229A; S96821A; S96822A; S96829A; S96921A; S96922A; S96929A
	Contusion, initial encounter	S0003XA; S0010XA; S0011XA; S0012XA; S0033XA; S00431A; S00432A; S00439A; S00531A; S00532A; S0083XA; S0093XA; S0510XA; S0511XA; S0512XA; S100XXA; S1083XA; S1093XA; S2000XA; S2001XA; S2002XA; S2020XA; S20211A; S20212A; S20219A; S20221A; S20222A; S20229A; S2601XA; S2611XA; S2691XA; S27321A; S27322A; S27329A; S27421A; S27422A; S27429A; S2752XA; S27802A; S27812A;

		S27892A; S300XXA; S301XXA; S30201A; S30202A; S3021XA; S3022XA; S3023XA; S303XXA; S36020A; S36021A; S36029A; S36112A; S36122A; S36220A; S36221A; S36222A; S36229A; S3632XA; S36420A; S36428A; S36429A; S36520A; S36521A; S36522A; S36523A; S36528A; S36529A; S3662XA; S36892A; S3692XA; S37011A; S37012A; S37019A; S37021A; S37022A; S37029A; S3712XA; S3722XA; S3732XA; S37421A; S37422A; S37429A; S37521A; S37522A; S37529A; S3762XA; S37812A; S37822A; S37892A; S3792XA; S40011A; S40012A; S40019A; S40021A; S40022A; S40029A; S5000XA; S5001XA; S5002XA; S5010XA; S5011XA; S5012XA; S6000XA; S60011A; S60012A; S60019A; S60021A; S60022A; S60029A; S60031A; S60032A; S60039A; S60041A; S60042A; S60049A; S60051A; S60052A; S60059A; S6010XA; S60111A; S60112A; S60119A; S60121A; S60122A; S60129A; S60131A; S60132A; S60139A; S60141A; S60142A; S60149A; S60151A; S60152A; S60159A; S60211A; S60212A; S60219A; S60221A; S60222A; S60229A; S7000XA; S7001XA; S7002XA; S7010XA; S7011XA; S7012XA; S8000XA; S8001XA; S8002XA; S8010XA; S8011XA; S8012XA; S9000XA; S9001XA; S9002XA; S90111A; S90112A; S90119A; S90121A; S90122A; S90129A; S90211A; S90212A; S90219A; S90221A; S90222A; S90229A; S9030XA; S9031XA; S9032XA
	Puncture, initial encounter	S0103XA; S0104XA; S01131A; S01132A; S01139A; S01141A; S01142A; S01149A; S0123XA; S0124XA; S01331A; S01332A; S01339A; S01341A; S01342A; S01349A; S01431A; S01432A; S01439A; S01441A; S01442A; S01449A; S01531A; S01532A; S01541A; S01542A; S0183XA; S0184XA; S0193XA; S0194XA; S11013A; S11014A; S11023A; S11024A; S11033A; S11034A; S1113XA; S1114XA; S1123XA; S1124XA; S1183XA; S1184XA; S1193XA; S1194XA; S21031A; S21032A; S21039A; S21041A; S21042A; S21049A; S21131A; S21132A; S21139A; S21141A; S21142A; S21149A; S21231A; S21232A; S21239A; S21241A; S21242A; S21249A; S21331A; S21332A; S21339A; S21341A; S21342A; S21349A; S21431A; S21432A; S21439A; S21441A; S21442A; S21449A; S2193XA; S2194XA; S31030A; S31031A; S31040A; S31041A; S31130A; S31131A; S31132A; S31133A; S31134A; S31135A; S31139A; S31140A; S31141A; S31142A; S31143A; S31144A; S31145A; S31149A; S3123XA; S3124XA; S3133XA; S3134XA; S3143XA; S3144XA; S31531A; S31532A; S31541A; S31542A; S31630A; S31631A; S31632A; S31633A; S31634A; S31635A;

		S31639A; S31640A; S31641A; S31642A; S31643A; S31644A; S31645A; S31649A; S31803A; S31804A; S31813A; S31814A; S31823A; S31824A; S31833A; S31834A; S41031A; S41032A; S41039A; S41041A; S41042A; S41049A; S41131A; S41132A; S41139A; S41141A; S41142A; S41149A; S51031A; S51032A; S51039A; S51041A; S51042A; S51049A; S51831A; S51832A; S51839A; S51841A; S51842A; S51849A; S61031A; S61032A; S61039A; S61041A; S61042A; S61049A; S61131A; S61132A; S61139A; S61141A; S61142A; S61149A; S61230A; S61231A; S61232A; S61233A; S61234A; S61235A; S61236A; S61237A; S61238A; S61239A; S61240A; S61241A; S61242A; S61243A; S61244A; S61245A; S61246A; S61247A; S61248A; S61249A; S61330A; S61331A; S61332A; S61333A; S61334A; S61335A; S61336A; S61337A; S61338A; S61339A; S61340A; S61341A; S61342A; S61343A; S61344A; S61345A; S61346A; S61347A; S61348A; S61349A; S61431A; S61432A; S61439A; S61441A; S61442A; S61449A; S61531A; S61532A; S61539A; S61541A; S61542A; S61549A; S71031A; S71032A; S71039A; S71041A; S71042A; S71049A; S71131A; S71132A; S71139A; S71141A; S71142A; S71149A; S81031A; S81032A; S81039A; S81041A; S81042A; S81049A; S81831A; S81832A; S81839A; S81841A; S81842A; S81849A; S91031A; S91032A; S91039A; S91041A; S91042A; S91049A; S91131A; S91132A; S91133A; S91134A; S91135A; S91136A; S91139A; S91141A; S91142A; S91143A; S91144A; S91145A; S91146A; S91149A; S91231A; S91232A; S91233A; S91234A; S91235A; S91236A; S91239A; S91241A; S91242A; S91243A; S91244A; S91245A; S91246A; S91249A; S91331A; S91332A; S91339A; S91341A; S91342A; S91349A
	Penetrating injuries, initial encounter	S0540XA; S0541XA; S0542XA; S0550XA; S0551XA; S0552XA; S0560XA; S0561XA; S0562XA
	Superficial injuries, initial encounter	S0000XA; S00201A; S00202A; S00209A; S0030XA; S00401A; S00402A; S00409A; S00501A; S00502A; S0080XA; S0090XA; S1010XA; S1080XA; S1090XA; S20101A; S20102A; S20109A; S20301A; S20302A; S20309A; S20401A; S20402A; S20409A; S2090XA; S3091XA; S3092XA; S3093XA; S3094XA; S3095XA; S3096XA; S3097XA; S3098XA; S40911A; S40912A; S40919A; S40921A; S40922A; S40929A; S50901A; S50902A; S50909A; S50911A; S50912A; S50919A; S60391A; S60392A; S60399A; S60911A; S60912A; S60919A; S60921A; S60922A; S60929A; S60931A; S60932A; S60939A; S60940A; S60941A; S60942A; S60943A; S60944A; S60945A; S60946A; S60947A; S60948A; S60949A; S70911A; S70912A; S70919A; S70921A; S70922A; S70929A; S80911A; S80912A; S80919A; S80921A; S80922A;

		S80929A; S90911A; S90912A; S90919A; S90921A; S90922A; S90929A; S90931A; S90932A; S90933A; S90934A; S90935A; S90936A
	Superficial foreign bodies, initial encounter	S0005XA; S00251A; S00252A; S00259A; S0035XA; S00451A; S00452A; S00459A; S00551A; S00552A; S0085XA; S0095XA; S1015XA; S1085XA; S1095XA; S20151A; S20152A; S20159A; S20351A; S20352A; S20359A; S20451A; S20452A; S20459A; S2095XA; S30850A; S30851A; S30852A; S30853A; S30854A; S30855A; S30856A; S30857A; S40251A; S40252A; S40259A; S40851A; S40852A; S40859A; S50351A; S50352A; S50359A; S50851A; S50852A; S50859A; S60351A; S60352A; S60359A; S60450A; S60451A; S60452A; S60453A; S60454A; S60455A; S60456A; S60457A; S60458A; S60459A; S60551A; S60552A; S60559A; S60851A; S60852A; S60859A; S70251A; S70252A; S70259A; S70351A; S70352A; S70359A; S80251A; S80252A; S80259A; S80851A; S80852A; S80859A; S90451A; S90452A; S90453A; S90454A; S90455A; S90456A; S90551A; S90552A; S90559A; S90851A; S90852A; S90859A
	Other open wounds, initial encounter	S0100XA; S01101A; S01102A; S01109A; S0120XA; S01301A; S01302A; S01309A; S01401A; S01402A; S01409A; S01501A; S01502A; S0180XA; S0190XA; S11019A; S11029A; S11039A; S1110XA; S1120XA; S1180XA; S1189XA; S1190XA; S21001A; S21002A; S21009A; S21101A; S21102A; S21109A; S21201A; S21202A; S21209A; S21301A; S21302A; S21309A; S21401A; S21402A; S21409A; S2190XA; S31000A; S31001A; S31100A; S31101A; S31102A; S31103A; S31104A; S31105A; S31109A; S3120XA; S3130XA; S3140XA; S31501A; S31502A; S31600A; S31601A; S31602A; S31603A; S31604A; S31605A; S31609A; S31809A; S31819A; S31829A; S31839A; S41001A; S41002A; S41009A; S41101A; S41102A; S41109A; S45301A; S45302A; S45309A; S45391A; S45392A; S45399A; S51001A; S51002A; S51009A; S51801A; S51802A; S51809A; S61001A; S61002A; S61009A; S61101A; S61102A; S61109A; S61200A; S61201A; S61202A; S61203A; S61204A; S61205A; S61206A; S61207A; S61208A; S61209A; S61300A; S61301A; S61302A; S61303A; S61304A; S61305A; S61306A; S61307A; S61308A; S61309A; S61401A; S61402A; S61409A; S61501A; S61502A; S61509A; S65201A; S65202A; S65209A; S65291A; S65292A; S65299A; S71001A; S71002A; S71009A; S71101A; S71102A; S71109A; S81001A; S81002A; S81009A; S81801A; S81802A; S81809A; S91001A; S91002A; S91009A; S91101A; S91102A; S91103A; S91104A; S91105A; S91106A; S91109A; S91201A;

		S91202A; S91203A; S91204A; S91205A; S91206A; S91209A; S91301A; S91302A; S91309A
Rule 3A	Major depressive disorders	F0631; F0632; F32; F320; F321; F322; F323; F324; F325; F328; F329; F33; F330; F331; F332; F333; F334; F3340; F3341; F3342; F338; F339; F4321; F4323
	Bipolar disorders	F31; F310; F311; F3110; F3111; F3112; F3113; F312; F313; F3130; F3131; F3132; F314; F315; F316; F3160; F3161; F3162; F3163; F3164; F317; F3170; F3171; F3172; F3173; F3174; F3175; F3176; F3177; F3178; F3181; F319
	Manic disorders	F0633; F30; F301; F3010; F3011; F3012; F3013; F302; F303; F304; F308; F309
	Other mood disorders	F063; F0630; F0631; F0632; F0633; F0634; F1014; F1024; F1094; F1114; F1124; F1194; F1314; F1324; F1394; F1414; F1424; F1494; F1514; F1524; F1594; F1614; F1624; F1694; F1814; F1824; F1894; F1914; F1924; F1994; F34; F348; F349; F39
	Dysthymia	F341
	Psychotic disorders	F060; F062; F1015; F10150; F10151; F10159; F1025; F10250; F10251; F10259; F1095; F10950; F10951; F10959; F1115; F11150; F11151; F11159; F1125; F11250; F11251; F11259; F1195; F11950; F11951; F11959; F1215; F12150; F12151; F12159; F1225; F12250; F12251; F12259; F1295; F12950; F12951; F12959; F1315; F13150; F13151; F13159; F1325; F13250; F13251; F13259; F1395; F13950; F13951; F13959; F1415; F14150; F14151; F14159; F1425; F14250; F14251; F14259; F1495; F14950; F14951; F14959; F1515; F15150; F15151; F15159; F1525; F15250; F15251; F15259; F1595; F15950; F15951; F15959; F1615; F16150; F16151; F16159; F1625; F16250; F16251; F16259; F1695; F16950; F16951; F16959; F1815; F18150; F18151; F18159; F1825; F18250; F18251; F18259; F1895; F18950; F18951; F18959; F1915; F19150; F19151; F19159; F1925; F19250; F19251; F19259; F1995; F19950; F19951; F19959; F23; F24; F28; F29; F48; F488; F489; F53
	Adjustment disorders	F43; F432; F4320; F4321; F4322; F4323; F4324; F4325; F4329
	Stress disorders	F430; F431; F4310; F4311; F4312; F438; F439

	Schizophrenic disorders	F20; F200; F201; F202; F203; F205; F208; F2081; F2089; F209; F21; F25; F250; F251; F258; F259; F601
	Other Delusional disorders	F22
	Primary hallucination disorders	R440; R441; R442; R443
	Personality disorders	F07; F070; F078; F0789; F079; F60; F600; F602; F603; F604; F605; F606; F607; F608; F6081; F6089; F609; F68; F688; F69
	Behavioral disturbances	F0151; F0281; F0391
	Unspecified mental disorder	F99
Rule 3B	Event, undetermined intent, initial encounter	Y210XXA; Y211XXA; Y212XXA; Y213XXA; Y214XXA; Y218XXA; Y219XXA; Y22XXXA; Y230XXA; Y231XXA; Y232XXA; Y233XXA; Y238XXA; Y239XXA; Y240XXA; Y248XXA; Y249XXA; Y25XXXA; Y26XXXA; Y270XXA; Y271XXA; Y272XXA; Y273XXA; Y278XXA; Y279XXA; Y280XXA; Y281XXA; Y282XXA; Y288XXA; Y289XXA; Y29XXXA; Y30XXXA; Y31XXXA; Y32XXXA; Y33XXXA
	Poison, undetermined intent, initial encounter	T360X4A; T361X4A; T362X4A; T363X4A; T364X4A; T365X4A; T366X4A; T367X4A; T368X4A; T3694XA; T370X4A; T371X4A; T372X4A; T373X4A; T374X4A; T375X4A; T378X4A; T3794XA; T380X4A; T381X4A; T382X4A; T383X4A; T384X4A; T385X4A; T386X4A; T387X4A; T38804A; T38814A; T38894A; T38904A; T38994A; T39014A; T39094A; T391X4A; T392X4A; T39314A; T39394A; T394X4A; T398X4A; T3994XA; T400X4A; T401X4A; T402X4A; T403X4A; T404X4A; T405X4A; T40604A; T40694A; T407X4A; T408X4A; T40904A; T40994A; T410X4A; T411X4A; T41204A; T41294A; T413X4A; T4144XA; T415X4A; T420X4A; T421X4A; T422X4A; T423X4A; T424X4A; T425X4A; T426X4A; T4274XA; T428X4A; T43014A; T43024A; T431X4A; T43204A; T43214A; T43224A; T43294A; T433X4A; T434X4A; T43504A; T43594A; T43604A; T43614A; T43624A; T43634A; T43694A; T438X4A; T4394XA; T440X4A; T441X4A; T442X4A; T443X4A; T444X4A; T445X4A; T446X4A; T447X4A; T448X4A; T44904A; T44994A; T450X4A; T451X4A; T452X4A; T453X4A; T454X4A; T45514A; T45524A; T45604A; T45614A; T45624A; T45694A; T457X4A; T458X4A;

		T4594XA; T460X4A; T461X4A; T462X4A; T463X4A; T464X4A; T465X4A; T466X4A; T467X4A; T468X4A; T46904A; T46994A; T470X4A; T471X4A; T472X4A; T473X4A; T474X4A; T475X4A; T476X4A; T477X4A; T478X4A; T4794XA; T480X4A; T481X4A; T48204A; T48294A; T483X4A; T484X4A; T485X4A; T486X4A; T48904A; T48994A; T490X4A; T491X4A; T492X4A; T493X4A; T494X4A; T495X4A; T496X4A; T497X4A; T498X4A; T4994XA; T500X4A; T501X4A; T502X4A; T503X4A; T504X4A; T505X4A; T506X4A; T507X4A; T508X4A; T50A14A; T50A24A; T50A94A; T50B14A; T50B94A; T50Z14A; T50Z94A; T50904A; T50994A
	Toxic effect, undetermined intent, initial encounter	T510X4A; T511X4A; T512X4A; T513X4A; T518X4A; T5194XA; T520X4A; T521X4A; T522X4A; T523X4A; T524X4A; T528X4A; T5294XA; T530X4A; T531X4A; T532X4A; T533X4A; T534X4A; T535X4A; T536X4A; T537X4A; T5394XA; T540X4A; T541X4A; T542X4A; T543X4A; T5494XA; T550X4A; T551X4A; T560X4A; T561X4A; T562X4A; T563X4A; T564X4A; T565X4A; T566X4A; T567X4A; T56814A; T56894A; T5694XA; T570X4A; T571X4A; T572X4A; T573X4A; T578X4A; T5794XA; T5804XA; T5814XA; T582X4A; T588X4A; T5894XA; T590X4A; T591X4A; T592X4A; T593X4A; T594X4A; T595X4A; T596X4A; T597X4A; T59814A; T59894A; T5994XA
	Asphyxiation, undetermined intent, initial encounter	T71114A; T71124A; T71134A; T71144A; T71154A; T71164A; T71194A; T71224A; T71234A
	Contusion, laceration, and hemorrhage, initial encounter	S06370A; S06371A; S06372A; S06373A; S06374A; S06375A; S06376A; S06377A; S06378A; S06379A
	Contusion and Laceration, initial encounter	S06310A; S06311A; S06312A; S06313A; S06314A; S06315A; S06316A; S06317A; S06318A; S06319A; S06320A; S06321A; S06322A; S06323A; S06324A; S06325A; S06326A; S06327A; S06328A; S06329A; S06330A; S06331A; S06332A; S06333A; S06334A; S06335A; S06336A; S06337A; S06338A; S06339A
	Laceration, initial encounter	S0101XA; S0102XA; S01111A; S01112A; S01119A; S01121A; S01122A; S01129A; S0121XA; S0122XA; S01311A; S01312A; S01319A; S01321A; S01322A; S01329A; S01411A; S01412A; S01419A; S01421A; S01422A; S01429A; S01511A; S01512A; S01521A; S01522A; S0181XA;

		S0182XA; S0191XA; S0192XA; S0520XA; S0521XA; S0522XA; S0530XA; S0531XA; S0532XA; S0912XA; S1181XA; S1182XA; S1191XA; S1192XA; S162XXA; S26020A; S26021A; S26022A; S2612XA; S2692XA; S36230A; S36240A; S36250A; S36260A; S46121A; S46122A; S46129A; S51811A; S51812A; S51819A; S51821A; S51822A; S51829A; S55011A; S55012A; S55019A; S55111A; S55112A; S55119A; S55211A; S55212A; S55219A; S55811A; S55812A; S55819A; S55911A; S55912A; S55919A; S56021A; S56022A; S56029A; S56121A; S56122A; S56123A; S56124A; S56125A; S56126A; S56127A; S56128A; S56129A; S56221A; S56222A; S56229A; S56321A; S56322A; S56329A; S56421A; S56422A; S56423A; S56424A; S56425A; S56426A; S56427A; S56428A; S56429A; S56521A; S56522A; S56529A; S56821A; S56822A; S56829A; S56921A; S56922A; S56929A; S61511A; S61512A; S61519A; S61521A; S61522A; S61529A; S65011A; S65012A; S65019A; S65111A; S65112A; S65119A; S65811A; S65812A; S65819A; S65911A; S65912A; S65919A; S66021A; S66022A; S66029A; S66120A; S66121A; S66122A; S66123A; S66124A; S66125A; S66126A; S66127A; S66128A; S66129A; S66221A; S66222A; S66229A; S66320A; S66321A; S66322A; S66323A; S66324A; S66325A; S66326A; S66327A; S66328A; S66329A; S66421A; S66422A; S66429A; S66520A; S66521A; S66522A; S66523A; S66524A; S66525A; S66526A; S66527A; S66528A; S66529A; S66821A; S66822A; S66829A; S66921A; S66922A; S66929A; S75011A; S75012A; S75019A; S75021A; S75022A; S75029A; S75111A; S75112A; S75119A; S75121A; S75122A; S75129A
	Contusion, initial encounter	S0003XA; S0010XA; S0011XA; S0012XA; S0033XA; S00431A; S00432A; S00439A; S00531A; S00532A; S0083XA; S0093XA; S0510XA; S0511XA; S0512XA; S1083XA; S1093XA; S2601XA; S2611XA; S2691XA; S36220A; S5010XA; S5011XA; S5012XA; S60211A; S60212A; S60219A
	Puncture, initial encounter	S0103XA; S0104XA; S01131A; S01132A; S01139A; S01141A; S01142A; S01149A; S0123XA; S0124XA; S01331A; S01332A; S01339A; S01341A; S01342A; S01349A; S01431A; S01432A; S01439A; S01441A; S01442A; S01449A; S01531A; S01532A; S01541A; S01542A; S0183XA; S0184XA; S0193XA; S0194XA; S1183XA; S1184XA; S1193XA; S1194XA; S51831A; S51832A; S51839A; S51841A; S51842A; S51849A; S61531A; S61532A; S61539A; S61541A; S61542A; S61549A

	Penetrating injuries, initial encounter	S0540XA; S0541XA; S0542XA; S0550XA; S0551XA; S0552XA; S0560XA; S0561XA; S0562XA
	Superficial injuries, initial encounter	S0000XA; S00201A; S00202A; S00209A; S0030XA; S00401A; S00402A; S00409A; S00501A; S00502A; S0080XA; S0090XA; S1080XA; S1090XA; S50911A; S50912A; S50919A; S60911A; S60912A; S60919A
	Superficial foreign bodies, initial encounter	S0005XA; S00251A; S00252A; S00259A; S0035XA; S00451A; S00452A; S00459A; S00551A; S00552A; S0085XA; S0095XA; S1085XA; S1095XA; S50851A; S50852A; S50859A; S60851A; S60852A; S60859A
	Other open wounds, initial encounter	S0100XA; S01101A; S01102A; S01109A; S0120XA; S01301A; S01302A; S01309A; S01401A; S01402A; S01409A; S01501A; S01502A; S0180XA; S0190XA; S1180XA; S1189XA; S1190XA; S51801A; S51802A; S51809A; S61501A; S61502A; S61509A
