# Supplementary material for: Feasibility and importance of universal suicide screening in a pediatric emergency department
Source: PLoS One. 2025 Jun 23;20(6):e0321934. doi: 10.1371/journal.pone.0321934 (PMC12184887; doi:10.1371/journal.pone.0321934)
Supplement: S1 Table — (PDF) [file pone.0321934.s002.pdf]

S1 Table. Comparisons of patient characteristics by risk level.

						Race and ethnicity									
		Age group		Gender		White		Black		Hispanic or Latino		Other race		Medicaid	
**Risk group 1**	**Risk group 2**	χ^2	***p***	χ^2	***p***	χ^2	***p***	χ^2	***p***	χ^2	***p***	χ^2	***p***	χ^2	***p***
**Minimal**	**Low**	49.9	<0.001	144.9	<0.001	4.6	0.19	0.2	1.00	1.9	>0.99	0.4	>0.99	8.6	0.02
	**Moderate**	31.2	<0.001	58.9	<0.001	17.5	<0.001	5.0	0.16	9.3	0.01	0.6	>0.99	0.6	>0.99
	**High**	53.0	<0.001	270.1	<0.001	65.7	<0.001	14.3	0.001	50.1	<0.001	6.5	0.07	17.9	<0.001
**Low**	**Moderate**	2.1	0.90	0.6	>0.99	6.5	0.06	3.1	0.46	4.2	0.25	1.1	>0.99	4.7	0.18
	**High**	<0.1	>0.99	11.9	0.003	18.2	<0.001	6.4	0.07	18.8	<0.001	5.3	0.13	27.7	<0.001
**Moderate**	**High**	1.8	>0.99	2.2	0.81	0.1	>0.99	<0.1	1.00	0.6	>0.99	0.2	>0.99	1.8	>0.99
