# Supplementary material for: Feasibility and importance of universal suicide screening in a pediatric emergency department
Source: PLoS One. 2025 Jun 23;20(6):e0321934. doi: 10.1371/journal.pone.0321934 (PMC12184887; doi:10.1371/journal.pone.0321934)
Supplement: S2 Table — (PDF) [file pone.0321934.s003.pdf]

S2 Table. Comparison of return rates in 6 months by risk level.

	**Returned in 6 months**				**Compared to**					
	**No**		**No**		**Low risk**		**Moderate risk**		**High risk**	
**Risk level**	**%**	***n***	**%**	***n***	χ^2	***p***	χ^2	***p***	χ^2	***p***
**Minimal risk**	40.2	5,837	59.8	8,696	8.4	0.02	0.1	>0.99	0.3	>0.99
**Low risk**	44.5	539	55.5	673	-	-	3.1	0.48	6.5	0.07
**Moderate risk**	39.1	144	60.9	224	-	-	-	-	<0.1	>0.99
**High risk**	39.3	479	60.7	740	-	-	-	-	-	-
